# Maintenance of xylem Network Transport Capacity: A Review of Embolism Repair in Vascular Plants

**DOI:** 10.3389/fpls.2013.00108

**Published:** 2013-04-24

**Authors:** Craig R. Brodersen, Andrew J. McElrone

**Affiliations:** ^1^Horticultural Sciences Department, Citrus Research and Education Center, University of FloridaLake Alfred, FL, USA; ^2^Crops Pathology and Genetics Research Unit, USDA-ARSDavis, CA, USA; ^3^Department of Viticulture and Enology, University of California DavisDavis, CA, USA

**Keywords:** embolism, xylem transport, drought tolerance, ecophysiology of plants, biophysics

## Abstract

Maintenance of long distance water transport in xylem is essential to plant health and productivity. Both biotic and abiotic environmental conditions lead to embolism formation within the xylem resulting in lost transport capacity and ultimately death. Plants exhibit a variety of strategies to either prevent or restore hydraulic capacity through cavitation resistance with specialized anatomy, replacement of compromised conduits with new growth, and a metabolically active embolism repair mechanism. In recent years, mounting evidence suggests that metabolically active cells surrounding the xylem conduits in some, but not all, species are capable of restoring hydraulic conductivity. This review summarizes our current understanding of the osmotically driven embolism repair mechanism, the known genetic and anatomical components related to embolism repair, rehydration pathways through the xylem, and the role of capacitance. Anatomical differences between functional plant groups may be one of the limiting factors that allow some plants to refill while others do not, but further investigations are necessary to fully understand this dynamic process. Finally, xylem networks should no longer be considered an assemblage of dead, empty conduits, but instead a metabolically active tissue finely tuned to respond to ever changing environmental cues.

The success of vascular land plants is intimately tied to the maintenance and functionality of the xylem network. Hydraulic failure within the xylem network can result in tissue damage, decreases in gas-exchange, and ultimately plant death. Not surprisingly, plants have evolved mechanisms to both avoid embolism formation and restore xylem transport capacity once embolism occurs. Research over the past three decades has greatly advanced our understanding of the physiology and biophysics of these processes (Zwieniecki and Holbrook, 2009; Nardini et al., 2011). Here, we summarize recent and historical studies on the subject of embolism repair and evaluate the mechanisms utilized by species known to restore hydraulic conductivity from both ecological and anatomical perspectives.

Drought-induced embolisms form when xylem sap tension reaches a critical threshold, where air is aspirated through pit membranes separating adjacent conduits, or gas bubbles spontaneously nucleate from dissolved gas in the xylem sap (Tyree and Zimmermann, 2002). Because of the negative pressure inside the xylem conduits, the gas rapidly expands and forces water into connected, neighboring conduits (Tyree and Zimmermann, 2002). Embolisms can also form in xylem vessels following freeze-thaw events, where crystallization of liquid water forces dissolved gas out of solution. Once the ice melts gas bubbles remain and later expand to fill the conduit when tension is subsequently applied to the xylem sap (Sperry et al., 1988). Wounding and pathogen infections can also result in the entry of air into the xylem network (Dimond, 1970; Tyree and Sperry, 1989; McElrone et al., 2008), all of which lead to non-functional xylem conduits that disrupt the transport and distribution of water and nutrients throughout the plant. Despite the negative physiological impacts, most plant species live with some proportion of their xylem rendered non-functional due to embolism (Choat et al., 2012).

Hydraulic dysfunction caused by embolism is such a strong selective pressure that plants have evolved xylem anatomy specifically to prevent embolism formation and spread (Brodribb and Holbrook, 2004). Woody plants utilize multiple strategies to prevent hydraulic dysfunction due to embolism. Hydraulic capacitance (i.e., stored water reserves held in secondary xylem) buffers against extreme tension in the xylem sap (Meinzer et al., 2009). With the aid of specialized pit membrane structures (e.g., Pittermann et al., 2005; Brodribb et al., 2012) xylem anatomy is largely responsible for the cavitation resistance in many species, allowing them to thrive in a diverse range of habitats with low water availability (Johnson et al., 2012). Successful colonization of xeric habitats by both angiosperms and gymnosperms has been linked to xylem highly resistant to embolism formation (Brodribb et al., 2012). Several studies have shown the diversity of stem sectoring in angiosperms, and xeric habitats tend to support species with increasingly dissected wood compared to mesic habitats (Ellmore et al., 2006; Zanne et al., 2006; Espino and Schenk, 2009; Martínez-Cabrera et al., 2009). By sectoring secondary xylem into discrete units that supply specific portions of the canopy, embolism spread between units is limited, thereby maintaining network function in the remainder of the xylem (Brodersen et al., 2013). This strategy comes at the cost of low lateral distribution of water and nutrients around the stem or trunk, but selective pressures have favored sectoring over xylem integration in many xeric habitats (Schenk et al., 2008). While embolism avoidance through anatomical adaptations appears to govern desiccation tolerance in many species, environmental conditions often push species beyond cavitation thresholds, thus requiring mechanisms to restore hydraulic transport capacity.

## Novel Refilling: New Insights into the Biophysics and Genetics of Embolism Repair

Hypotheses describing the mechanism involved in embolism repair have long suggested that water droplets form on vessel walls and grow until the lumen refills. However, until recently, we lacked clear evidence demonstrating this process in xylem under tension (reviewed in Clearwater and Goldstein, 2005; Table 1). The mechanisms behind this process, however, have been widely hypothesized (see Nardini et al., 2011 for a full review) but largely unstudied due to insufficient resolution of non-destructive visualization tools. Beginning with the nuclear magnetic resonance (NMR) imaging work of Holbrook et al. (2001), the dynamics of embolism repair *in planta* started to unfold, and were followed by a number of studies that expanded the number of species and tissues in which embolism repair could be visualized (Windt et al., 2006; Scheenen et al., 2007). Brodersen et al. (2010) utilized high-resolution X-ray computed tomography (HRCT) to visualize this process *in planta*, in three dimensions, and with a short temporal resolution (ca. 30 min per scan). Their HRCT imaging analysis showed water droplet formation and growth in grapevine vessels where the bulk xylem tissue was under tension, as well as the dissolution of trapped gas bubbles within the xylem sap. These findings support similar images of droplets on vessels walls obtained using cryo-SEM that had not been fully validated as the result of refilling activity (Canny, 1997; Utsumi et al., 1998; Tyree et al., 1999). Water droplets originated from vessel walls were most closely oriented with the vasicentric axial parenchyma cells, thereby supporting the idea that living cells play a central role in embolism repair (Brodersen et al., 2010). The spatial patterns of embolism repair observed by Brodersen et al. (2010) also suggests that xylem tension can be decreased in refilling tissue while nearby (but unconnected) functional vessels remain under tension (i.e., not likely the result of root pressure). Patterns of water droplet formation closely resemble those of tyloses, which are known to arise from axial parenchyma, particularly the vasicentric axial parenchyma in angiosperm wood (Sun et al., 2008). Therefore, the paratracheal parenchyma cells appear to have two important roles in the functional status of the xylem conduits: to restore hydraulic conductivity in embolized conduits and to permanently seal off conduits with tyloses to prevent further pathogen or air spread throughout the vasculature. Further work is needed to explore how the cells perceive, interpret, and rapidly respond to varying stress signals, and what cues are required for the switch between embolism repair and tylose formation.

Clearwater and Goldstein (2005) questioned whether refilling is restricted to particular species, organs, or even in specific locations within the xylem. Given the recent work by Bucci et al. (2012) that provides additional support for the concept of hydraulic segmentation (i.e., petioles are more susceptible to cavitation than stems), improved capacity to restore hydraulic conductivity would be needed to respond to regular cycles of cavitation in this frequently stressed link of the flow pathway. The proximity to the photosynthetic mechanism would also supply ample sugars if the refilling mechanism were driven osmotically (argued most recently by Perrone et al., 2012). The role of xylary chloroplasts in the maintenance of hydraulic conductivity further supports the osmotic repair theory, and suggests that the process may be light-dependent in some species and tissues (Schmitz et al., 2012a). Thus, it is still unclear whether xylem in roots, trunks, stems, petioles, and leaf veins refill equally, but the differences in xylem organization between these tissues in plants suggests that the refilling mechanisms may be different depending on the location of the tissue (e.g., refilling in roots may be more strongly influenced by root pressure, while tissue higher in the canopy would rely more on “novel” refilling to restore hydraulic conductivity). Work is needed to evaluate whether refilling occurs similarly in different organs within the same plant (roots, stems, petioles). Is refilling in petioles of woody plants more similar to patterns seen in herbaceous species lacking secondary growth?

Vessel refilling rates have been found to be variable across individual plants of the same species and dependent on individual plant water status prior to refilling (Holbrook et al., 2001; Hacke and Sperry, 2003; Brodersen et al., 2010). Much of this variability may be related to local water supplies in the vicinity of the refilling vessels. In severely stressed plants, it may take much longer to initiate refilling because sufficient water needed to raise tissue water potentials must travel over longer distances along regions with low water potential that draw some of this water. Nardini et al. (2011) and Tyree et al. (1999) suggested that water needed for refilling ultimately arrives from the phloem. Sugars needed as osmoticum for refilling would be unloaded in the direction of embolized vessels via ray parenchyma. Those carbohydrates are then thought to be converted into simple, low-molecular weight sugars, transported across the membrane on to the conduit wall, thereby establishing a gradient to drive water movement away from the phloem (Nardini et al., 2011).

**Table 1:** **List of representative studies providing evidence for and against the restoration of hydraulic conductivity**.

Species	Family	Functional group	Refilling type	Study period	Method	Axial parenchyma	Tyloses	Embolism repair citation	Axial parenchyma citation	Tyloses citation
**EVIDENCE FOR EMBOLISM REPAIR**										
*Acer rubrum*	Aceraceae	A-D	N	H	PLC, dye	78v	No, gums	Zwieniecki and Holbrook (1998)	IW	Saitoh et al. (1993)
*Acer pseudoplatanus*	Aceraceae	A-D	N	M	PLC			Hacke and Sauter (1996)		
*Acer saccharum*	Aceraceae	A-D	RP	M	PLC			Sperry et al. (1988)		
*Betula corifolia*	Betulaceae	A-D	RP	M	PLC			Sperry (1993)		
*Betula pendula*	Betulaceae	A-D	N, RP	H, M	PLC, NMR	76, 77	Yes in *Betula*, plugs	Hacke and Sauter (1996), Westhoff et al. (2008)	IW	IW, Bauch et al. (1980)
*Betula platyphylla*	Betulaceae	A-D	N	H	Cryo-SEM, PLC	76	Yes in *Betula*, plugs	Utsumi et al. (1998), Ogasa et al. (2010)	IW	InsideWood (2004)
*Blepharocalyx salicifolius*	Myrtaceae	A-D	N, RP	H	PLC	77 in *B. gigantea*		Domec et al. (2006)	IW	
*Caryocar brasiliense*	Caryocaraceae	A-D	N	H	PLC	78 in *C. glabrum*; 78 in *C. villosum*	Yes in *Caryocar*	Bucci et al. (2003)	IW	IW
*Ceratonia siliqua*	Fabaceae	A-D	N	M, H	PLC	79, 80, 81, 83		Lo Gullo et al. (2003)	IW	
*Celastrus orbiculatus*	Celastraceae	A-D	RP	M	PLC	78, 79	Yes	Tibbetts and Ewers (2000)	IW	IW
*Cucumis sativus*	Cucurbitaceae	A-D	N	H	NMR	Yes	Yes	Scheenen et al. (2007)	Schweingruber (2011)	Jordan (1903)
*Fagopyrum esculentum*	Polygonaceae	A-D	N	H	Cryo-SEM		Yes in Polygonaceae	Buchard et al. (1999)		Foster (1967), IW
*Fagus sylvatica*	Fagaceae	A-D	N, RG	M	PLC	76	Yes	Hacke and Sauter (1996), Cochard et al. (2001)	IW	Foster (1967), IW
*Fraxinus americana*	Oleaceae	A-D	N, RG	H	PLC, dye	79, 80	Yes	Zwieniecki and Holbrook (1998)	IW	IW
*Glycine max*	Fabaceae	A-D	N	H	Cryo-SEM	No		Buchard et al. (1999)		Shimaji (1952)
*Helianthus annuus*	Asteraceae	A-D	N	H, M	Cryo-SEM; kmax	No	Yes in Asteraceae	Canny (1997), Buchard et al. (1999), Nardini et al. (2008), Stiller and Sperry (2002), Trifilò et al. (2003)		Biggs (1987)
*Juglans regia*	Juglandaceae	A-D	RP	M	Gen., carb	77	Yes	Sakr et al. (2003)	IW	
*Laurus nobilis*	Lauraceae	A-D	N	M, H, M	PLC	78, 79	No in Lauraceae	Salleo et al. (1996, 2004, 2006), Tyree et al. (1999), Hacke and Sperry (2003)	IW, Salleo et al. (2004)	Evert (2006)
*Lycopersicon esculentum*	Cucurbitaceae	A-D	N	M, H	PLC	No	Yes	Secchi et al. (2013)		Charest et al. (1984)
*Passiflora coerulea*	Passifloraceae	A-D	N	H, D	Cryo-SEM	79, 80, 81, 82, 83	Yes in *Passiflora*	Canny et al. (2007)	IW	
*Populus alba*	Salicaceae	A-D	N	D	PLC	75v, 89	Yes in *Populus*	Leng et al. (2012)	IW	Kitin et al. (2010)
*Populus nigra*	Salicaceae	A-D	N	H, D	Sap	75v, 89	Yes	Secchi and Zwieniecki (2012)	IW	Kitin et al. (2010)
*Populus trichocarpa*	Salicaceae	A-D	N	H, D	PLC	75v, 89	Yes	Secchi and Zwieniecki (2010), Secchi et al. (2011)	IW	Kitin et al. (2010)
*Prionostemma aspera*	Celastraceae	A-D	N	H, D	SWP, PLC	Yes		Johnson et al. (2013)	IW	
*Rhizophora mangle*	Rhizophoraceae	A-D	N	H	Cryo-SEM, PLC	78, 79	Yes	Melcher et al. (2001)	IW	Schmitz et al. (2012b)
*Salix sachalinensis*	Salicaceae	A-D	N	M	PLC, cryo-SEM	75v, 89	Yes	Utsumi et al. (1998)	IW	Saitoh et al. (1993)
*Sambucus nigra*	Adoxaceae	A-D	N, RG	M	PLC, cryo-SEM	75, 76, 78	Yes	Vogt (2001)	IW	IW
*Schefflera macrocarpa*	Araliaceae	A-D	N	H	PLC	75 in *Schefflera*	Yes in *Schefflera*	Bucci et al. (2003)	IW	Oskolski (1995)
*Simarouba glauca*	Simaroubiaceae	A-D	N	H	PLC	80, 82, 83		Brodribb and Holbrook (2004)	IW	
*Smilax rotundifolia*	Smilacaceae	A-D	N, RP	M	PLC			Cobb et al. (2007)		
*Sorbus aucuparia*	Rosaceae	A-D	N, RG	M	PLC	76, 77, 78	No	Vogt (2001)	IW	Saitoh et al. (1993), Evert (2006)
*Trichostigma octandrum*	Phytolaccaceae	A-D	N	H	SWP, PLC	No		Johnson et al. (2013)		
*Vitis spp*.	Vitaceae	A-D	N, RP	H, D	NMR; PLC; HRCT	78, 79	Yes	Sperry et al. (1987), Tibbetts and Ewers (2000), Holbrook et al. (2001), Brodersen et al. (2010), Zufferey et al. (2011), Kaldenhoff et al. (2008)	IW	Evert (2006), Sun et al. (2008)
*Zea mays*	Poaceae	A-M	RP	H, D	NMR, cryo-SEM	No		Tyree et al. (1986), Kaufmann et al. (2009), McCully (1999)		
*Oryza sativa*	Poaceae	A-M	N	H	PLC	No	Yes in Poaceae	Stiller et al. (2005)		Chaffey and Pearson (1985)
*Phyllostachys bambusoides*	Poaceae	A-M	N	M	HRCT (2D)	No	Yes in *Phyllostachys*	Lee and Kim (2008)		IW, Andre (1998), Liese and Weiner (1996)
*Rhapis excelsa*	Arecaceae	A-M	RP	H	PLC	No		Sperry (1986)		
*Rhipidocladum racemiflorum*	Poaceae	A-M	RP	M	PLC	No	Yes in Poaceae	Cochard et al. (1994)		Chaffey and Pearson (1985)
*Sinarundinaria nitida*	Poaceae	A-M	RP	H	PLC	No	Yes (Poaceae, bamboo)	Yang et al. (2012)		
*Abies balsamea*	Pinaceae	G		M	PLC			Sperry (1993)		
*Abies grandis*	Pinaceae	G	N, RG	M	PLC	No		McCulloh et al. (2011)		
*Picea rubens*	Pinaceae	G	N, RG	H, M	PLC, dye	No		Sperry (1993), Zwieniecki and Holbrook (1998)		
*Pinus contorta*	Pinaceae	G	N, RG	D	VWC	No	Yes in *Pinus*	Canny et al. (2007)		Peters (1974)
*Pinus sylvestris*	Pinaceae	G	N, RG	D, M	PLC	No	Yes in *Pinus*	Sobrado et al. (1992)		Peters (1974)
*Pseudotsuga menziesii*	Pinaceae	G	N, RG	M	PLC	No	Yes	McCulloh et al. (2011)		Kitin et al. (2010)
*Thuja plicata*	Cupressaceae	G	N, RG	M	PLC	No	No	McCulloh et al. (2011)		
*Tsuga heterophylla*	Pinaceae	G	N, RG	M	PLC	No	Yes in *T. canadensis*	McCulloh et al. (2011)		Biggs (1987)
**EVIDENCE AGAINST EMBOLISM REPAIR**										
*Acer negundo*	Aceraceae	A-D		H	PLC	78, 79	No, gums	Hacke and Sperry (2003)	IW	Saitoh et al. (1993), IW
*Actinidia spp*.	Actinidiaceae	A-D		H, D	NMR	77, 78, 79	Yes in *Actinidia*	Clearwater and Clark (2003)	IW	Renzi et al. (2012)
*Fagus grandifolia*	Fagaceae	A-D		M	PLC			Sperry (1993)		IW
*Quercus seratta*	Fagaceae	A-D		H	PLC	79	Yes in *Quercus*	Ogasa et al. (2010)	IW	Cochard and Tyree (1990)
*Ripogonum*	Ripogonaceae	A-M		H, D	NMR			Clearwater and Clark (2003)		

*A-D, dicot angiosperm; A-M, monocot angiosperm; G, gymnosperm; N, novel refilling; RG, regrowth; RP, root pressure; Mi, minutes; H, hours; D, days; Mo, months; PLC, percent loss of conductivity; Dye, dye infiltrations; NMR, nuclear magnetic resonance imaging; Cryo-SEM, cryogenic scanning electron microscopy; Gen., genetic screening of xylem sap; Carb., carbohydrate analysis of xylem sap; HRCT (2D), two dimensional high-resolution x-ray computed tomography imaging; HRCT (3D), three dimensional high-resolution x-ray, computed tomography imaging; VWC, volumetric water content; Sap, sapflow sensors; IW, InsideWood database (Wheeler, 2011); v, variable; 75, axial parenchyma absent or extremely rare; 76, axial parenchyma diffuse; 77, axial parenhyma diffuse-in-aggregates; 78, axial parenchyma scanty paratracheal; 79, axial parenchyma vasicentric; 80, axial parenchyma aliform; 81, axial parenchyma lozenge-aliform; 82, axial parenchyma winged-aliform; 83, axial parenchyma confluent*.

Alternatively, the remaining functional vessels or fibers could be providing the water needed for refilling. Producing vessels of different sizes should be advantageous compared to wood with a homogenous vessel diameter distribution. This strategy would ensure that large, cavitation prone vessels are in close proximity to smaller diameter, cavitation resistant vessels that could serve as a local water reservoir to aid in refilling or mitigate drought-induced xylem tension (Davis et al., 1999; Pittermann and Sperry, 2003). Heterogeneous vessel diameter distribution is common in woody plants, particularly in ring-porous species where seasonal water availability and tree growth influence vessel diameters (Carlquist, 2001), but these traits have yet to be fully explored with respect to embolism repair. Even under severe water stress grapevines still contain many water-filled vessels (Brodersen et al., 2013). In this case, water needed for refilling would travel in the remaining functional conduits to the proximity of the embolized xylem tissue and then follow more localized gradients to the parenchyma cells surrounding a cavitated conduit. The utilization of remaining functional vessels for water delivery would enable the bulk tissue to recover much more rapidly. A potential limitation of this theory is that continued transpiration may be required to move water through the remaining functional conduits. As increasingly more conduits cavitate, the supply of water continues to decline, which could contribute to “runaway cavitation” suggested by Tyree and Sperry (1988).

Salleo et al. (2009) and Nardini et al. (2011) concluded that starch to sugar conversion was an integral part of embolism recovery in *Laurus nobilis*. Depletion of the carbohydrate reserves from xylem parenchyma cells creates a strong sink signal for phloem unloading that triggers movement of sugar and water in a radial orientation via the rays. Starch granules are readily apparent in the fibers of grapevine xylem (Brodersen et al., 2010), and unlike fibers in most other species, those in grapevines, shrubs, and other lianas are often found with living protoplasts at maturity (Fahn and Leshem, 1963). The combination of a plentiful carbohydrate source and presumably active metabolic capacity in fiber cells suggests that they may play a more active role in embolism repair than previously thought (Nardini et al., 2011). Several studies have documented a reduction in starch content upon embolism formation in xylem (Salleo et al., 2009; Secchi and Zwieniecki, 2011), and repeated cycles of embolism formation and repair may rapidly deplete these reserves and disable the refilling mechanism after repeated drought stress. Such depletion would ultimately lead to carbon starvation and the inability for plants to recover from prolonged or repeated drought events (McDowell et al., 2008), although the debate over carbon starvation theory is ongoing (Sala, 2009; Sala et al., 2010, 2012; McDowell, 2011). It has yet to be determined how the products of starch hydrolysis move from the sites of storage in various xylem cells to the vessel associated parenchyma. Clearly, the spatial resolution of these responses across cells needs to be explored in greater detail.

Secchi and colleagues have completed much recent work to elucidate the mechanism of refilling in *Populus trichocarpa* stems (Secchi and Zwieniecki, 2010, 2011, 2012; Secchi et al., 2011). Gene expression analyses from water stressed plants revealed an up-regulation of ion transport, aquaporins, and carbon metabolism (Secchi et al., 2011; Secchi and Zwieniecki, 2012). These responses sync with chemical analysis of the fluid from non-functional vessels that exhibit lower pH (presumably due to proton pump activity causing an efflux of H+ into the vessel lumen and associated with putative sucrose-cation co-transporter activity) and increased cations and sugar levels. Perrone et al. (2012) found similar patterns in grapevine refilling petioles, where carbon metabolism and aquaporin expression was strongly up-regulated. Up-regulation of carbohydrate (including disaccharide) metabolism (presumably associated with starch degradation) in embolized stems would promote the release of sucrose that could be used as the source of energy needed to support refilling through increased respiration or as an osmoticum. Buildup of osmoticum in the vessel lumen would trigger an efflux of water that is facilitated by the up-regulation of aquaporins (PIPs and TIPs). Whether the localization of aquaporins within axial parenchyma surrounding conduits changes during refilling (i.e., asymmetric distribution) to direct flow is yet to be determined.

To date, most studies investigating the genetic components of embolism formation and repair have depended on bulk tissue analysis. To further elucidate the anatomical pathways involved in water and carbohydrate transport among the various cells types research is needed to study the chemical and genetic changes occurring in the individual components along the pathway. This will be particularly important if we are to glean general response patterns across species with differing xylem anatomy (i.e., amount of vessel associated parenchyma vs. fibers vs. parenchyma rays) and the orientation of the cells and the contents (i.e., starch) in various locations. The probability of conduit refilling is therefore dependent on the storage and metabolic activity of the cells immediately surrounding the conduit, the length of the pathway between the axial parenchyma cells and the rays, and the distance from the rays to the phloem.

## Emerging Trends in Angiosperm and Gymnosperm Embolism Repair

Compiling evidence for or against embolism repair is complicated by the wide variety of methods, species, and temporal resolution of the studies. Assembled in Table 1 is a representative list of the species for which some type of embolism repair or restoration of hydraulic conductivity has been reported. The 47 species represent 26 different plant families and include angiosperms (both monocots and dicots) and gymnosperms. There are no overwhelming trends that would suggest a unifying theory to explain embolism repair in all vascular plants, but a few intriguing patterns could serve as the basis for directions of future research (Table 1). In some cases the data are contradictory: species within the same genus (e.g., Acer; Zwieniecki and Holbrook, 1998; Hacke and Sperry, 2003) or functional group (e.g., lianas, *Vitis*, and *Actinidia*) show evidence both for and against embolism repair.

From this collection of studies, several trends warrant further investigation based on current theory (Table 1). First, the axial parenchyma believed to be involved in the osmotic mechanism for embolism repair are prominent in most woody angiosperms that have been shown to refill. In the few cases where axial parenchyma are not present, those species are herbaceous annual plants without secondary xylem, and often show evidence of root pressure in the refilling process (e.g., *Zea mays*, Kaufmann et al., 2009). Second, for monocotyledonous species where refilling has been reported, most of the species are thought to have refilled via root pressure, although five of the six species fall within Poaceae, limiting the scope of these findings at this time. However, root pressure appears to be common in grass species, and among bamboo species total plant height increases linearly with basal root pressure (Cao et al., 2012). Monocots lack secondary xylem and axial parenchyma that are implicated in the osmotic embolism repair mechanism (Andre, 1998). Selection for root pressure in these species solves the embolism repair problem and negates the need for carbohydrate transport along the pathway common in woody angiosperms.

Not surprisingly it appears as though anatomy plays a significant role in the rate of embolism repair and how it is accomplished. Dicots with secondary xylem appear to be the most efficient at refilling (typically achieved in minutes, hours, or days). In monocots and other species refilling can also be rapid (i.e., minutes or hours), which might be more closely related to the short stature of the study species; root pressure would not have to transport water over great distances or overcome significant gravitational resistances. *Rhapis excelsa* (Sperry, 1986), a palm tree, is the only exception among the monocots where conduits were able to refill rapidly over the course of approximately 6 h when detached from the trunk (i.e., without root pressure). So while monocots may utilize root pressure to refill embolized conduits, this mechanism is not exclusive to or universal within this group (Ewers et al., 1997; Fisher et al., 1997), and only a few woody angiosperms appear to utilize root pressure, typically in the recovery from freeze-thaw induced embolism (Sperry et al., 1987; Cochard et al., 2001; Ewers et al., 2001).

To date, *Vitis vinifera* is one of the fastest reported refilling species. The combination of both “novel” refilling aided by axial parenchyma and root pressure likely allows *V. vinifera* to efficiently repair dysfunctional xylem (Sperry et al., 1987; Isnard and Silk, 2009) and maintain water transport despite daily cycles of embolism formation (Schultz, 2003) and susceptibility to freeze-thaw embolism during spring conditions (Sperry et al., 1987). While the combination of both “novel” and root pressure refilling have yet to be simultaneously visualized, different parts of the plant likely utilize one mechanism more than another based on the proximity to the root system and the capacity of the roots to produce pressure (Cao et al., 2012). Root pressure is more likely to occur in the trunk due to the proximity of the wood to the root system, whereas “novel” refilling is known to occur in stems (Holbrook et al., 2001; Brodersen et al., 2010). Some species, but not all, likely utilize a combination of these mechanisms to maintain xylem functionality at different points of the year depending on environmental conditions (e.g., temperature, photoperiod, and water availability).

In conifers with no axial parenchyma restoration of hydraulic conductivity in branches or trunks typically occurred over the course of days or months. In a few cases, small stems and needles were shown to recover from hydraulic losses within hours (Zwieniecki and Holbrook, 1998; McCulloh et al., 2011). Johnson et al. (2012) showed that conifer needles could go through cycles of embolism and repair over the course of a single day. One component to successful embolism repair in needles may be water absorption through the cuticle (Limm et al., 2009; Simonin et al., 2009). Cuticular absorption would reduce the tension on the xylem, and minimize the demand for water in needles high in the canopy, and that reduced tension would eventually be translated to the wood in the trunk. Embolism formation is likely one of the limitations to tree height (Koch et al., 2004; Burgess and Dawson, 2007) Because gravity sets limits on water potential gradients, maximum xylem water potentials that are necessary for refilling are unlikely to occur in the upper canopies of very tall trees, with the exception of cloud immersion events where foliar absorption could mitigate stem water potential enough to allow for refilling (Burgess and Dawson, 2007; Limm et al., 2009).

In conifer needles, phloem and tracheids are located in close proximity, and the tracheids are also flanked by transfusion tissue, which have been implicated by Canny (1993) as a source of solutes from the transpiration stream. This tissue is a combination of tracheids and living parenchyma, and can empty without generating detectable acoustic emissions (i.e., cavitation) at low hydrostatic pressure, presumably to supply the remaining tracheids with water or mitigate internal pressure gradients (Johnson et al., 2009). The translocation of carbohydrates for refilling in stems and trunks of conifer wood would be largely dependent on the ray tissue that is interspersed between the tracheids. If refilling in conifers is dependent on carbohydrate delivery to cells surrounding the conduits, then the tracheids in direct contact with the rays would likely refill first, and the process would be much slower for tracheids farther from the rays. This distance vs. refilling ability relationship in conifers should play a significant role in the speed at which refilling can occur and the total cross sectional area of the conduits that are able to refill. However until more *in planta* studies are performed with conifers the spatial and temporal dynamics of embolism repair will likely remain elusive.

An alternative hypothesis for conifers is that instead of employing a refilling mechanism, these trees simply replace non-functional xylem with new growth, an idea that seems to be supported by conifer wood anatomy (Brodribb et al., 2010). Recent data suggests that some sort of novel refilling is employed by stems and trunks of conifers, however, the story is less clear. McCulloh et al. (2011) showed restoration of hydraulic conductivity in four conifer species over a growing season, and their data suggest that new growth was not enough to account for the gains in recovery, particularly since much of the recovery was complete before cambial activity began in the spring. Further, the functional sapwood was many rings deep into the tree, and the new growth would only account for a small portion of the gains in hydraulic conductivity. They argue that some form of “novel” refilling plays a role in conifer wood because of the small diameter of the tracheids, the negative tensions observed on the xylem sap, and the height at which the experiments were performed likely rules out embolism dissolution by way of positive pressure via the biophysics of La Place’s law (Yang and Tyree, 1992).

Our analysis of the literature also identified an additional five species representing four different plant families from four separate studies that did not appear to refill (Table 1). The data for these species are difficult to interpret, and it is unclear whether the species involved are unable to refill or whether the environmental conditions for plants in the field were not conducive to embolism repair. For example, Hacke and Sauter (1996) showed refilling in *Populus balsamifera* 1 year, but refilling was slow and incomplete the following year in the same species. Such experiments highlight the inherent variability of the refilling mechanism, which is likely dependent on the physiological health of the trees involved in the experiments. As such, repeated measurements on trees grown in a controlled environment will often be necessary to establish whether or not some species refill, which can then be complemented by field studies to better understand the dynamics of embolism repair in nature.

## Conflict of Interest Statement

The authors declare that the research was conducted in the absence of any commercial or financial relationships that could be construed as a potential conflict of interest.
